# 3D bioprinting via an *in situ* crosslinking technique towards engineering cartilage tissue

**DOI:** 10.1038/s41598-019-56117-3

**Published:** 2019-12-27

**Authors:** Jonathan H. Galarraga, Mi Y. Kwon, Jason A. Burdick

**Affiliations:** Department of Bioengineering, University of Pennsylvania, Philadelphia, PA 19104 USA

**Keywords:** Adult stem cells, Musculoskeletal system, Biomaterials

## Abstract

3D bioprinting is a promising approach for the repair of cartilage tissue after damage due to injury or disease; however, the design of 3D printed scaffolds has been limited by the availability of bioinks with requisite printability, cytocompatibility, and bioactivity. To address this, we developed an approach termed *in situ* crosslinking that permits the printing of non-viscous, photocrosslinkable bioinks via the direct-curing of the bioink with light through a photopermeable capillary prior to deposition. Using a norbornene-modified hyaluronic acid (NorHA) macromer as a representative bioink and our understanding of thiol-ene curing kinetics with visible light, we varied the printing parameters (e.g., capillary length, flow rate, light intensity) to identify printing conditions that were optimal for the ink. The printing process was cytocompatible, with high cell viability and homogenous distribution of mesenchymal stromal cells (MSCs) observed throughout printed constructs. Over 56 days of culture in chondrogenic media, printed constructs increased in compressive moduli, biochemical content (i.e., sulfated glycosaminoglycans, collagen), and histological staining of matrix associated with cartilage tissue. This generalizable printing approach may be used towards the repair of focal defects in articular cartilage or broadly towards widespread biomedical applications across a range of photocrosslinkable bioinks that can now be printed.

## Introduction

Cartilage is a load-bearing connective tissue found in articulating joints that permits movement between bones with minimal friction. When articular cartilage is damaged due to disease or traumatic injury, loss of cartilage throughout the joint surface may occur, resulting in reduced joint mobility and eventually osteoarthritis^1^. Since native cartilage does not possess any regenerative capacity, surgical interventions are often required to mitigate the progression of cartilage degeneration in afflicted patients. Procedures such as microfracture aim to recruit cells (e.g., mesenchymal stromal cells, MSCs) from the underlying bone marrow, while cell-based therapies such as matrix-assisted autologous chondrocyte implantation (MACI) focus on scaffolds to elicit tissue formation from donor cells^2^. Despite their clinical use, these approaches have only exhibited limited success, as they fail to fully restore the function of healthy cartilage. These findings have motivated the use of tissue engineering to improve the quality of repair cartilage for clinical applications.

Within the field of tissue engineering, 3D bioprinting enables the fabrication of cell-laden hydrogel scaffolds with anatomically relevant structures and patient-specific geometries, improving the prospects for repair tissue integration^3^. For example, poly(ethylene glycol) (PEG)-based hydrogel implants with embedded chondrocytes have been fabricated via extrusion-based bioprinting and shown to integrate with adjacent cartilage tissue in *ex vivo* osteochondral plugs^4^. Unlike alternative fabrication approaches such as micromolding, 3D bioprinting permits the modular and scalable design of precise scaffold features that better recapitulate properties of native tissue. Specifically, 3D bioprinting allows for unparalleled spatial control over materials^5,6^ or cell types^7^ in 3D space, which has been used to mimic the zonal stratification of properties found in cartilage or osteochondral units^8^. Daly *et al*. used the inkjet printing of cell spheroids into 3D printed polycaprolactone (PCL)-based microchambers for guidance of spheroid growth and fusion, permitting the formation of neotissues with depth-dependent collagen architectures^9^. PCL has also been utilized to increase the mechanics of printed hydrogels (e.g., fibrin-collagen, alginate, agarose, PEG) with embedded chondrocytes or MSCs towards cartilage formation^10–13^, including through the combination of melt electrowriting of PCL with extrusion-based printing of gelatin-methacryloyl (GelMA)^14^. Other hydrogel inks that have been previously used for engineering cartilage include hyaluronic acid (HA)^7^, decellularized extracellular matrix (ECM)^15^, and gellan gum^7,16^.

Bioinks, which are typically comprised of a hydrogel precursor solution containing cells^17^, must exhibit a number of requisite design specifications to be printable with traditional printing technologies. For example, in extrusion-based 3D bioprinting, bioinks must first have suitable rheological properties such that they can readily flow through a printer head. If a candidate bioink is too viscous, appreciable shear forces will be exerted on encapsulated cells, reducing cell viability and long-term functional properties of printed constructs. Beyond flow, bioinks must also possess sufficient mechanical integrity upon deposition so that extruded filaments are stable and can be deposited in a layer-by-layer manner. A number of bioinks have been designed with these specific criteria in mind, such as with guest-host supramolecular hydrogels that are shear-thinning and self-healing and can be stabilized via secondary covalent crosslinking^18^. However, if a bioink is non-viscous, it will flow rapidly upon deposition due to gravity, limiting printed filament resolution.

While many advances have been made in the design and implementation of bioinks, including towards cartilage tissue engineering, it is of interest to expand on the possible properties available with printable bioinks rather than only using inks that meet current printing criteria. As described by Malda *et al*., the traditional window for bioprinting is often not optimal for maintaining desired cell behavior, including cell viability^19^. Further, it may be of interest to harness diverse bioink properties, as it is now well known that biochemical and biophysical properties of hydrogels influence encapsulated cells - for example, the presentation of signaling cues such as ECM ligands and mechanics are known to regulate cell differentiation, proliferation and migration^20^. Thus, generalizable techniques that allow the printing of a wider range of bioinks are of interest for tissue engineering to introduce optimal cellular environments.

To overcome the challenges of printing bioinks that do not meet traditional criteria, a number of strategies have been pursued. One approach involves the introduction of rheological additives, such as silicates^21–23^ or nanocellulose^24,25^ into bioinks to impart desired rheological properties for extrusion-based printing. Support hydrogels have also been developed, where hydrogels can be printed in any arbitrary space, allowing for embedded printing of geometries not feasible by traditional layer-by-layer fabrication. For example, hydrogels have been printed into self-healing, supramolecular guest-host hydrogels^26^ and into granular support baths comprised of either a gelatin slurry^27^ or Carbopol microgels^28^. Sacrificial materials have also been utilized, where polymers such as alginate can be introduced into an ink for stabilization (e.g., via calcium through a coaxial needle) and then later washed away after the desired ink material is stabilized, such as with photocrosslinking^29^. Lastly, jammed microgels have recently been used for printing, as many materials can be formed into microgels and jammed to meet printing requirements, including with encapsulated cells^30^. While each of these approaches expands upon the number of candidate bioinks available, the need for additives or post-processing steps could impede or compromise the design of target cellular microenvironments.

**Figure 1:**
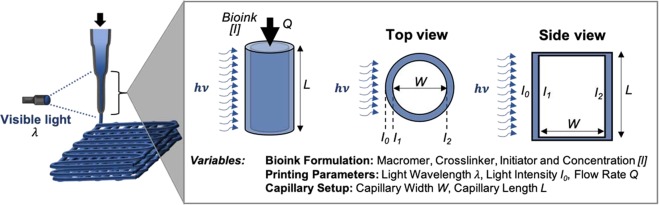
Schematic of *in situ* crosslinking approach for 3D bioprinting. Bioinks are loaded into a syringe and irradiated with light through a photopermeable capillary during extrusion, resulting in the plug flow of filaments through the end of the capillary. There are numerous variables within the printing approach, including the bioink formulation, the printing parameters, and the capillary setup, all of which can influence printing success. These should be balanced to regulate the residence time of the bioink within the light path (*Q*, *L*, *W*), as well as the reaction kinetics of crosslinking (*[I]*, *I*_0_). The intensity of light across the capillary lumen varies as a function of light attenuation due to the capillary walls and absorbing species within the designed bioink.

In the context of photocrosslinkable bioinks, we recently developed an approach to print non-viscous polymers, where light exposure occurs prior to bioink deposition as it passes through a photopermeable capillary (Fig. 1, Supplementary Fig. 1)^31^. With this *in situ* crosslinking approach, stable hydrogel filaments are readily extruded across many hydrogel types, while the shear forces generated on cells are attenuated so that high cell viability is conserved. Furthermore, this printing approach does not require post-processing steps or the use of rheological additives, allowing for one-step 3D printing of bioactive materials. Here, we selected one potential bioink of interest for the 3D bioprinting of cartilage tissue, based on norbornene-modified hyaluronic acid (NorHA)^32^ that can be crosslinked via a thiol-ene reaction in the presence of visible light and a water-soluble photoinitiator^33^. HA is a promising biomaterial in cartilage tissue engineering, particularly towards influencing MSC chondrogenesis^34–36^; however, the NorHA bioink is non-viscous and does not meet traditional printing requirements. In this study, we explain the various steps used to implement *in situ* crosslinking with this NorHA bioink and illustrate its utility in engineering cartilage with encapsulated MSCs.

## Results

### Design of *in situ* crosslinking approach based on bioink formulation

**Figure 2:**
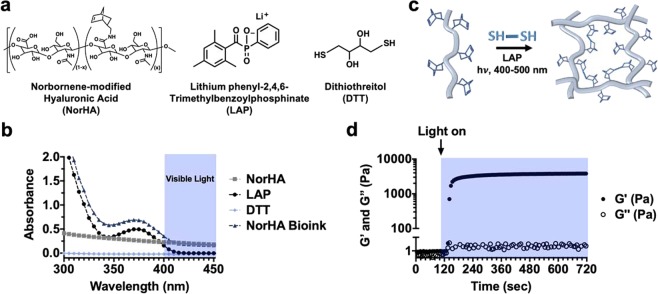
NorHA bioink composition and crosslinking. (**a**) Chemical structures of components incorporated into NorHA bioinks and their (**b**) absorption spectra, including for NorHA (2 wt%, square), LAP (0.05 wt%, circle), DTT (0.08 wt%, diamond), and their combination into a single bioink formulation (triangle). (**c**) Schematic of thiol-ene reaction employed to crosslink the NorHA bioink in the presence of visible light and LAP photoinitiator. (**d**) Representative photorheology time sweep (1 Hz, 0.5% strain) during the photocrosslinking of the NorHA bioink with visible light (400–500 nm) at *I*_1_ = 10 mW/cm^2^, illustrating increases in storage (G′, closed circles) and loss (G″, open circles) moduli over time.

HA was modified with pendant norbornene functional groups, such that approximately 40% of disaccharide repeat units contained norbornene (NorHA), as determined by quantitative ^1^H NMR (Supplementary Fig. 2). Bioinks were formulated from 2 wt% NorHA, 0.05 wt% LAP, and 0.08 wt% DTT (Fig. 2a). To assess how much light each ink component attenuates, the absorption spectra of NorHA, LAP and DTT were measured from 300–500 nm (Fig. 2b). After elucidating each of these respective absorption spectra, the molar extinction coefficients (${\epsilon }$) of ink components were determined using Beer-Lambert Law (Eq. (1)), which states that the absorption of a species of interest is proportional to the pathlength of light (*W*), the concentration of the species (*c*), and the degree to which the species absorbs that specific wavelength of light (${\epsilon }$).

1 $$A={\epsilon }Wc$$

As shown in these spectra, the degree of light attenuation due to DTT within the bioink is negligible, whereas both NorHA and LAP absorb light up to ~420 nm. To better understand the potential for light attenuation through the printer’s photopermeable capillary, the maximum amount of attenuation possible, which occurs at 400 nm, was quantified. Since ${\epsilon }$ can be determined using Eq. (1) and absorbance measurements of NorHA and LAP samples with known concentrations, the molar extinction coefficient for LAP at 400 nm was determined to be ~0.078 cm^−1^mM^−1^, while the coefficient for NorHA was ~855 cm^−1^mM^−1^. The light attenuation (of 400 nm light) due to multiple absorbing species can then be quantified via an alternative form of Beer-Lambert law, given by Eq. (2).

2 $$I={I}_{0}{e}^{-W({{\epsilon }}_{I}[I]+{{\epsilon }}_{NorHA}[NorHA])}$$

Thus, the drop in light intensity across the capillary lumen (*W* = 800 μm) due to the bioink used in our printing setup was negligible (Supplementary Fig. 1), as the initial intensity within the capillary (*I*_1_) only decreases ~3% across the width of the capillary (*I*_2_); however, larger decreases in light intensity could be expected if a higher concentration of initiator (*[I]*), wider tubing (increased *W*), or different wavelength (*λ*) of light were employed (Supplementary Fig. 3). Finally, to target a specific *I*_1_ within the photopermeable capillary, experimental relationships of light attenuation due to the capillary walls themselves were developed (Supplementary Fig. 4).

### Photorheology to identify permissible printing regimes

The NorHA within the bioink undergoes a thiol-ene reaction for crosslinking (Fig. 2c), which can be monitored experimentally with photorheology to assess the kinetics of gelation for our distinct ink formulation (Fig. 2d). Photorheology time sweeps were performed at *I*_2_ ~ 4.86, 9.72 and 14.6 mW/cm^2^ (corresponding to *I*_1_ = 5, 10 and 15 mW/cm^2^, respectively) towards creating gelation profiles that could predict permissible printing regimes (Fig. 2d, Supplementary Fig. 5). When NorHA inks were initially subjected to shear at 1 Hz and 0.5% strain, the storage (G′) and loss (G″) moduli were on the order of 1–10 Pa, consistent with a non-viscous material. It was not possible to measure the viscosity of the initial bioink formulation. However, upon irradiation with visible light, a rapid evolution of mechanics was observed (increasing G′), indicating NorHA crosslinking into an elastic hydrogel.

These photorheological time sweeps were normalized to their maximum value to develop a heuristic for the time required for G′ to plateau; it has previously been shown that the percent of maximum storage G′ correlates with the conversion of crosslinker in thiol-ene reactions^37^. This metric was therefore used to quantitatively estimate the extent of reaction as a function of time. Since the capillary length, bioink volumetric flow rate, and incident light intensity are all user-defined parameters for *in situ* crosslinking, we aimed to elucidate how each of these variables can be tuned in conjunction with these normalized gelation profiles to enhance ink printability.

**Figure 3:**
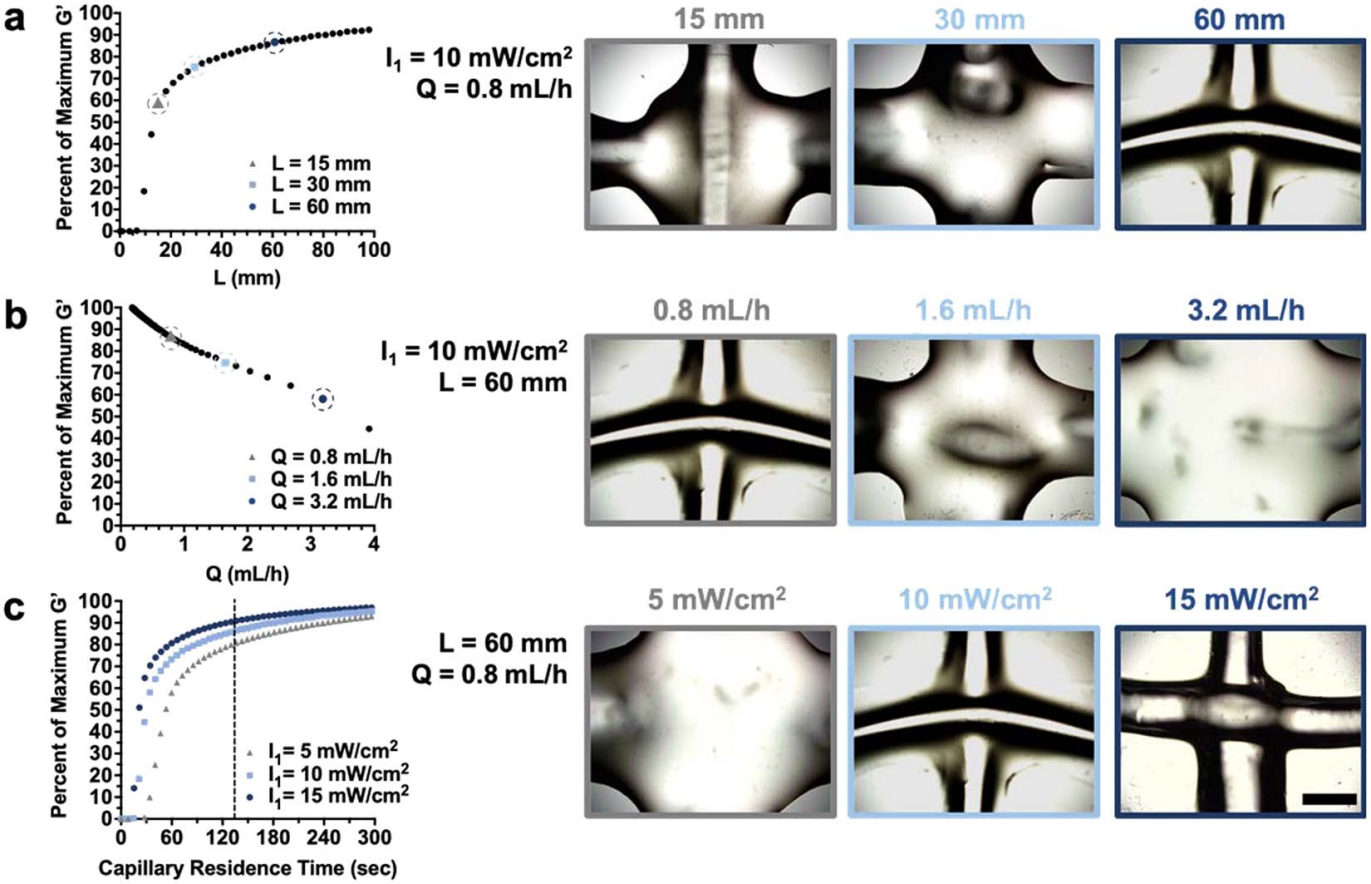
Identification of permissible printing conditions via photorheology. (**a**) Left: Percent of maximum G′ as a function of variable capillary lengths and Right: representative images of overlaying filaments, with *I*_1_ = 10 mW/cm^2^, *Q* = 0.8 mL/h and variable capillary lengths (*L* = 15, 30, 60 mm). (**b**) Left: Percent of maximum G′ as a function of variable flow rates and Right: representative images of overlaying filaments, with *L* = 60 mm, *I*_1_ = 10 mW/cm^2^ and variable flow rates (*Q* = 0.8, 1.6, 3.2 mL/h). (**c**) Left: Percent of maximum G′ as a function of capillary residence time across variable light intensities (dashed line indicates the fixed residence time of 135 seconds) and Right: representative images of overlaying filaments, with *L* = 60 mm, *Q* = 0.8 mL/h and variable light intensities (*I*_1_ = 5, 10, 15 mW/cm^2^). Scale bar = 1 mm. Note: the same representative image was used for the printing parameters used subsequently in this study (*L* = 60 mm, *Q* = 0.8 mL/h, *I*_1_ = 10 mW/cm^2^).

First, an analysis was performed on the influence of capillary lengths on ink printability, while setting the light intensity and flow rate at constant values (*I*_1_ = 10 mW/cm^2^, *Q* = 0.8 mL/h). If the time of light exposure (Fig. 2d; x-axis) is multiplied by the ink velocity (which is set by the flow rate and the width of the capillary lumen), then a relationship between the percent of maximum G′ versus capillary length can be generated (Fig. 3a). By experimentally printing the bioink under various conditions, it is clear that the quality of printed filaments is dependent on the capillary length. Here, a capillary length of 60 mm was needed for good print resolution, whereas capillary lengths of 15 mm and 30 mm resulted in irregular and spread filaments, indicating that the curing was not complete.

Similarly, these gelation profiles can be employed towards understanding how bioink flow rate influences the *in situ* crosslinking process, while setting the light intensity and capillary length at constant values (*I*_1_ = 10 mW/cm^2^, *L* = 60 mm). A relationship between the percent of maximum G′ versus bioink flow rate was obtained (Fig. 3b) by converting the time of light exposure (Fig. 2d; x-axis) into volumetric flow rate using Eq. (3) below, where *W* = 0.8 mm for this experiment.

3 $$Q=\frac{\pi \ast L\ast {W}^{2}}{4\ast time}$$

Again, the NorHA bioink was printed with varied bioink flow rates to observe the influence of printing conditions on filament quality (Fig. 3b). Here a flow rate as slow as 0.8 mL/h was needed for high resolution filaments, as faster flow rates did not permit sufficient times for bioink curing under this *in situ* crosslinking setup and resulted in spread filaments.

Finally, the influence of light intensity on crosslinking was explored, where increased light intensities led to more rapid curing (Fig. 3c). While selecting a common ink residence time of 135 seconds (Fig. 3c; *L* = 60 mm, *Q* = 0.8 mL/h), it was clear that at least 10 mW/cm^2^ light intensity was needed for filament curing, whereas lower light intensities were not sufficient for crosslinking under the specific *in situ* crosslinking setup. Overall, the most consistently printable and stable filaments were achieved when printing conditions resulted in NorHA bioinks reaching >85% of their maximum G′. It should be noted that the maximum G′ achieved after 10 minutes of irradiation may decrease appreciably if the reaction kinetics are slow (i.e., significantly lower light intensities); therefore, the predictive power of these gelation profiles is only valid if a plateau in storage modulus is observed in the photorheology studies.

**Figure 4:**
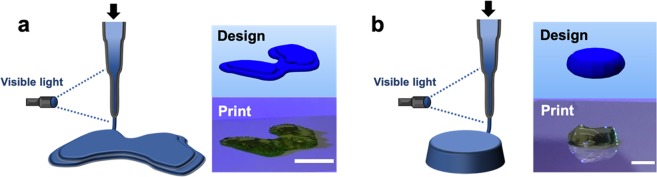
Representative multi-layered constructs printed via *in situ* crosslinking. Left: Schematic of *in situ* crosslinking method and Right: CAD design and representative image of a printed construct (labeled with food coloring) for designs of (**a**) a model femoral condyle or (**b**) a disc (~1.5 mm thickness, ~6.5 mm diameter). Scale bars = 1 cm (**a**) and 5 mm (**b**).

Through the implementation of this approach, a set of optimal printing conditions was determined (*L* = 60 min, *Q* = 0.8 mL/h, *I*_1_ = 10 mW/cm^2^) and utilized to print larger, multilayered constructs. Specifically, *in situ* crosslinking was employed to create large constructs with anatomically relevant features, such as a femoral condyle (Fig. 4a, Supplementary Video 1). In addition, discs (~1.5 mm thickness, ~6.5 mm diameter) were printed (Fig. 4b) and shown to retain their structure after immersion in PBS for one week (Supplementary Fig. 6). To demonstrate the reproducibility of this printing approach, we quantified the percent error between the targeted and observed dimensions of printed filaments and discs, which both exhibited on average ~3% error (Supplementary Fig. 7). To ensure the viability of this printing approach towards fabricating constructs for long-term culture and neocartilage formation, we also validated that the printing process does not alter the swelling behavior or the mechanics of NorHA hydrogels (Supplementary Fig. 8). Specifically, the volumetric swelling ratios and compressive moduli of both printed and casted discs incubated in PBS were determined at 0, 1, 3, and 7 days, and no differences were observed across these timepoints.

### 3D bioprinting with *in situ* crosslinking of NorHA bioink for MSC encapsulation

**Figure 5:**
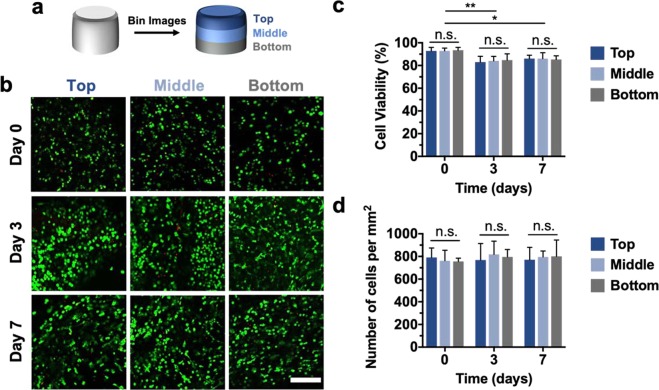
Cell viability and distribution in printed constructs. (**a**) Schematic demonstrating the binning of acquired Live/Dead confocal images for analysis of the top, middle, and bottom thirds of printed discs. (**b**) Representative Live/Dead images (scale bar = 200 μm), (**c**) quantification of cell viability, and (**d**) quantification of cell density for the top, middle, and bottom thirds of printed discs after 0, 3, and 7 days of culture. n ≥ 3, *p < 0.05, **p < 0.01, n.s. = not significant.

To assess the cytocompatibility of the printing process, primary juvenile bovine MSCs were isolated, printed into discs, and cultured in chondrogenic media for up to one week. Confocal images of constructs stained with Live/Dead assays indicated that high cell viabilities (>85%) persisted through 7 days after printing, although small decreases in viability were observed from the initial time point (day 0) to 3 and 7 days. To ensure that the observed cytocompatibility was conserved throughout all depths of the printed constructs, confocal images for distinct thirds (top, middle, bottom) of each disc were analyzed (Fig. 5a,b). At all timepoints (days 0, 3, 7), cell viabilities in distinct regions of the discs exhibited no significant differences, indicating that large constructs could be readily printed while retaining consistent cell viability throughout the duration of printing (Fig. 5c).

One challenge in the printing of bioinks is cell sedimentation and achieving a homogenous distribution of cells throughout a printed construct^38^. Thus, cell densities were also quantified throughout different depths of the printed discs to demonstrate that cell settling did not impact cell distribution at the print times employed with the *in situ* crosslinking technique. At each timepoint, the cell density was within the range of 750–820 cells/mm^2^, with no significant differences existing between different depths of the constructs or across different timepoints (Fig. 5d). Therefore, *in situ* crosslinking supported the fabrication of multi-layered constructs with viable and well-distributed MSCs.

### Neocartilage formation in 3D printed NorHA constructs

After validating the printability and cytocompatibility of NorHA hydrogels printed via *in situ* crosslinking, we next printed constructs for long-term culture to investigate neocartilage formation. Printed discs were cultured for up to 56 days in chondrogenic media; upon fixing, all samples were characterized to assess changes in biochemical content, mechanics, and matrix distribution over time. Initially, printed discs were analyzed after three days of culture via PCR to ensure that encapsulated MSCs would undergo chondrogenesis (Supplementary Fig. 9); the observed expression of chondrogenic markers such as type II-collagen (COLII), aggrecan (ACAN), and SOX9 indicated that printed constructs were conducive to neocartilage formation.

**Figure 6:**
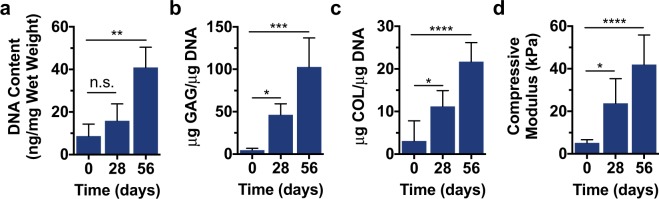
Mechanical characterization and biochemical analysis of printed constructs. (**a**) DNA content, (**b**) sulfated glycosaminoglycan (GAG) content, (**c**) collagen (COL) content, and (**d**) compressive moduli for printed constructs after 0, 28, and 56 days of culture. n ≥ 3, *p < 0.05, **p < 0.01, ***p < 0.001, ****p < 0.0001, n.s. = not significant.

After 56 days of culture, printed discs exhibited an increase in normalized DNA content, suggesting that viable cells proliferated and persisted throughout the duration of culture (Fig. 6a). Further evidence of neocartilage formation is provided by metrics of increased sulfated glycosaminoglycan (GAG) and collagen (COL) contents (Fig. 6b,c). Both of these extracellular matrix components are indicative of MSC chondrogenesis and tissue maturation, demonstrating that printed discs formed into neocartilage. Sulfated GAG content increased to over 100 μg/μg DNA by 56 days, likely enhancing the mechanics of the printed constructs, as these polysaccharides impart osmotic swelling and high compressive properties to native tissue^39^. Collagen, the main ECM-protein found in cartilage, was also deposited by embedded cells, with collagen content increasing 7-fold from 0 to 56 days. These results were corroborated by dynamic mechanical analysis, which showed increases in the compressive moduli of printed discs from 5.2 ± 1.5 kPa initially to 42.0 ± 13.9 kPa after 56 days of culture (Fig. 6d). Although these mechanics pale in comparison to those of native bovine articular cartilage, which has been shown to possess Young’s moduli on the order of 0.3–0.6 MPa^40^ and aggregate moduli ranging between 0.5 MPa and 1.0 MPa^41^, the observed increases in compressive moduli demonstrate the evolution of functional tissue properties in printed constructs.

**Figure 7:**
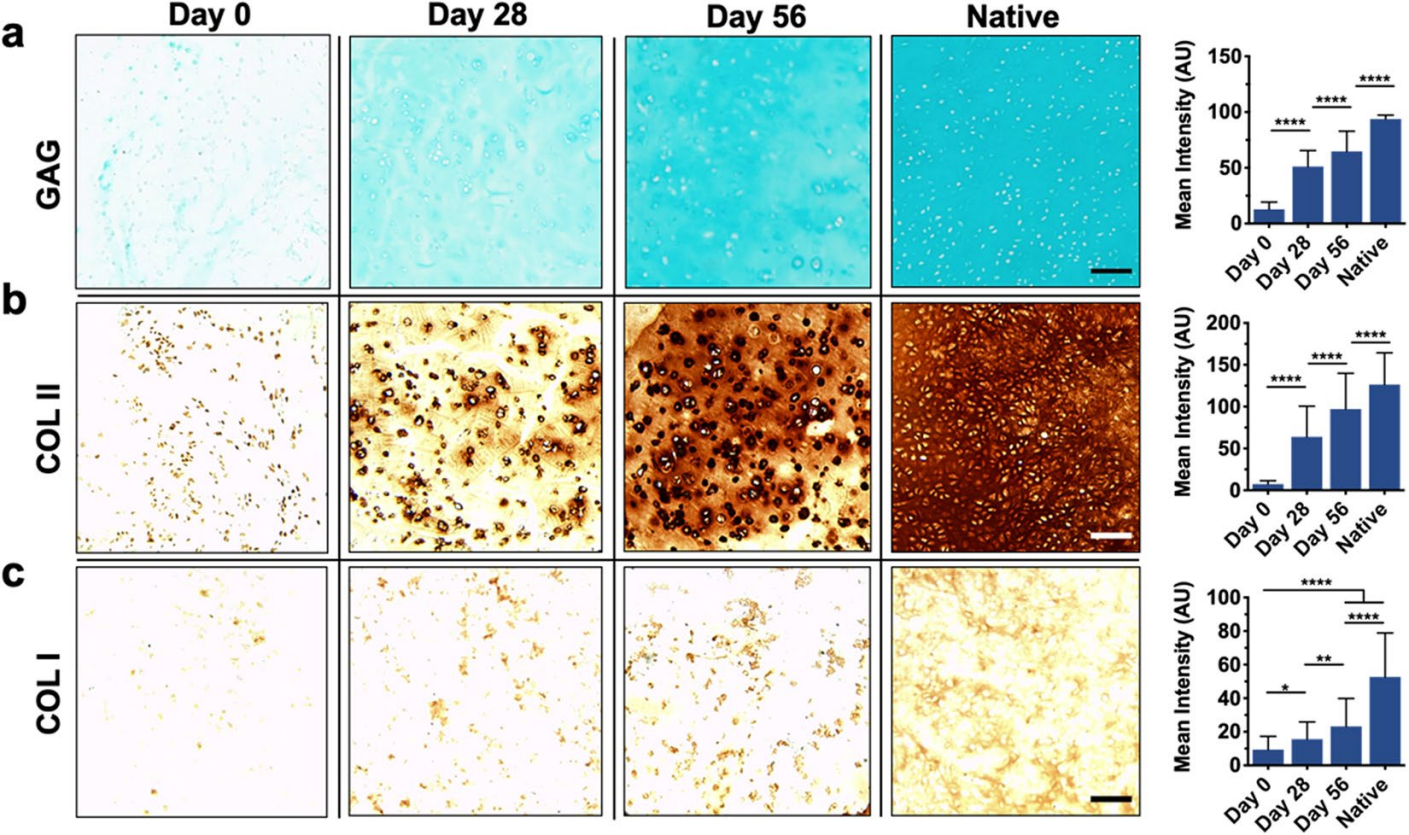
Histological evaluation of printed constructs. Left: Representative images and Right: staining quantification of (**a**) alcian blue staining for sulfated glycosaminoglycans (GAG), (**b**) immunohistochemistry for type II collagen (COL II), and (**c**) immunohistochemistry for type I collagen (COL I) for printed constructs after 0, 28, and 56 days of culture or native bovine articular cartilage. Scale bars = 100 μm, n ≥ 15 sections, 45 images per group, *p < 0.05, **p < 0.01, ****p < 0.0001.

Histological analyses were subsequently performed to assess the distribution of ECM components within the printed discs. Alcian blue staining indicated that GAGs were homogenously distributed by encapsulated MSCs by as early as 28 days, with staining intensities increasing over time and trending towards native tissue levels (Fig. 7a). Collagen II (COLII), one of the most abundant matrix proteins found in cartilage, was also detected in printed constructs, indicating that appreciable matrix was formed over long-term culture (Fig. 7b). The observed increases in COL II staining intensity are of interest, as COLII imparts tensile strength to cartilage in native tissue^39^. Furthermore, the deposition of COLII in printed discs was disperse and well distributed, albeit less homogenous than the observed GAGs. Noticeably, COLII staining was most intense at 56 days within the pericellular space of encapsulated cells. Finally, the distribution of collagen I (COL I), which is more prevalent in fibrocartilage, was observed to qualitatively assess the phenotype of the fabricated neocartilage (Fig. 7c). While increases in COLI staining were observed from the initial timepoint to 56 days, there was appreciably less COL I than COLII in printed constructs, suggesting that the tissue formed more closely resembles hyaline cartilage over fibrocartilage.

**Figure 8:**
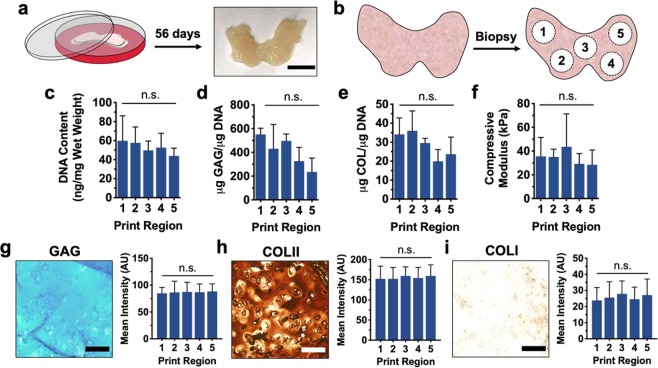
Culture and characterization of printed femoral condyles. (**a**) Schematic of printed femoral condyle and image of printed construct after 56 days of culture. (**b**) Schematic of five distinct print regions biopsied from printed femoral condyle models for analysis. (**c**) DNA content, (**d**) sulfated glycosaminoglycan (GAG) content, (**e**) collagen (COL) content, and (**f**) compressive moduli for construct biopsies after 56 days of culture. Left: Representative images and Right: staining quantification of (**g**) alcian blue staining for sulfated glycosaminoglycans (GAG), (**h**) immunohistochemistry for type II collagen (COL II), and (**i**) immunohistochemistry for type I collagen (COL I) for construct biopsies after 56 days of culture. Scale bars = 1 cm (**a**) and 100 μm (**g–i**), n = 3 printed constructs, n ≥ 15 sections, 45 images per group, n.s. = not significant.

It is noteworthy that this *in situ* crosslinking technique may also be leveraged towards the design and fabrication of neocartilage into more complex geometries. To this end, femoral condyles were printed and cultured for 56 days in a similar manner to printed discs (Fig. 8a), resulting in the formation of larger tissue constructs. To assess the homogeneity and quality of neotissue formed in these constructs, condyles were biopsied such that 4 mm discs were isolated from five distinct print regions (Fig. 8b). As anticipated, each of these biopsies exhibited biochemical content associated with neocartilage, including elevated amounts of normalized DNA content (Fig. 8c), sulfated GAG content (Fig. 8d) and collagen content (Fig. 8e). Tissue samples isolated from printed condyles also showed enhanced compressive properties relative to acellular constructs (Fig. 8f, Supplementary Fig. 8). It should be noted that any discrepancies observed between the moduli of biopsied tissue samples (i.e. from printed femoral condyles) and previously printed discs may be attributed to differences in sample topography, as the biopsied condyle samples possessed a convex surface. Interestingly, no significant differences in biochemical content or compressive moduli were observed across the five biopsied print regions of femoral condyles, suggesting that *in situ* crosslinking supports the fabrication of neocartilage in a controlled and scalable manner. Similarly, all five biopsied print regions displayed an appreciable amount of ECM deposition, as demonstrated by histological analysis (Fig. 8g–i). Staining intensities for GAG, COLII and COLI did not vary significantly between distinct print regions, and the relative amounts of COLII and COLI observed suggest that femoral condyle models were successfully printed to form hyaline cartilage.

## Discussion

To engineer precise tissues for clinical medicine, the development of scaffolds with complex, hierarchical structures are of great interest, particularly with patient-specific defect geometries^42^. 3D bioprinting is a promising approach towards this, including for the repair of cartilage^3,43^; however, the design of 3D bioprinted scaffolds has been limited to only a small number of bioinks with the requisite properties for printability. This inherently limits 3D bioprinting in tissue repair, as cells are responsive to their local environment and we would like to print materials to guide cell behavior, rather than use materials only because they are printable. To address this, we recently developed an approach that permits the printing of non-viscous, photocrosslinkable bioinks without the use of additives or sacrificial materials^31^.

Our *in situ* crosslinking approach is simple - the non-viscous bioink is cured with light as it passes through a photopermeable capillary, prior to deposition onto a surface (Fig. 1, Supplementary Fig. 1). The design of the system includes many variables that can be balanced to ensure crosslinking as the hydrogel precursor transits through the capillary; thus, it is important to understand both the reaction kinetics of the specific bioink composition and the residence time of the material within the capillary. The steps to *in situ* crosslinking include: (i) selecting a desired bioink (macromer, crosslinker, initiator/concentration), (ii) characterizing the gelation behavior for this bioink using the light wavelength and intensity available for the printing setup, and (iii) designing the capillary (width/length) and bioink flow rate for crosslinking to occur prior to deposition. For example, as the bioink’s residence time within the capillary increases (e.g., increased capillary length, lower volumetric flow rate), the light exposure time and time permitted for *in situ* crosslinking increases, resulting in elevated crosslinking until maximum conversions are reached. Similarly, increased reaction rates (e.g., increased initiator concentration or light intensity) increase the rate of gelation and support altered printing setups (e.g., shorter capillary lengths). Attention should be given to the exposure of cells to any harmful components (radicals, shear forces), but the photoencapsulation of cells and extrusion of cells from needles has now been performed extensively, and any issues are mitigated by following general considerations of these prior studies^44–46^. Too much curing during printing should also be considered, as it may lead to clogging of the capillary during the curing process.

HA-based hydrogels are of particular interest in cartilage repair since HA is a natural component of cartilage, biodegradable, non-toxic, and may be easily modified to form hydrogels with a range of properties; in addition, HA is already used in numerous clinical applications, such as in joint viscosupplements or tissue-fillers^47^. Therefore, NorHA was selected as a photocrosslinkable macromer to comprise our bioink for engineering cartilage. NorHA is crosslinked into hydrogels via a thiol-ene reaction, where radical species are first generated (e.g., light exposure of a photoinitiator) to subsequently form reactive thiyl radical intermediates in the presence of thiol-containing molecules; these intermediates may then undergo reactions with free norbornene groups^33^. Here, the photoinitiator LAP was selected since it is a water-soluble, visible light photoinitiator that has limited cytotoxicity and has been previously employed towards the formation of HA-based hydrogels^48^. Although visible light is used in this approach, macromer solutions were still stable under ambient light and the process can be used across a wide range of wavelengths with the appropriate initiator systems. Further, DTT was selected as the di-thiol crosslinker due to previous use in cell encapsulation^49^.

To implement this *in situ* crosslinking approach, careful consideration must first be given to the distinct components incorporated into the bioink (Fig. 2a). NorHA was used at a relatively low concentration (2 wt%), as it has been previously shown that lower crosslink densities give rise to hydrogels with increased nutrient transport and ECM dispersion by encapsulated cells^35^. Similarly, the concentration of LAP (0.05 wt%) was selected to ensure appreciably quick gelation kinetics while mitigating any potential cytotoxic effects. Varying DTT concentration has been shown to modulate the mechanics of NorHA hydrogels, as the degree of crosslinking is dependent on the number of crosslinks formed^32^; thus, 0.08 wt% DTT (ca. 5.2 mM) was used in the identified bioink to obtain gels with compressive moduli of approximately 6 kPa. The light absorbance of the bioink is dependent upon the selection of these components and their concentrations; thus, we characterized absorbance to understand both radical generation and potential light transmittance across the capillary. Significant light attenuation can alter the uniformity of reaction across the capillary and should be minimized where possible to reduce filament heterogeneity. To address this, quantitative Beer-Lambert Law calculations were performed to determine how light intensity varies during printing as a function of light wavelength, ink formulation, and capillary width (Supplementary Fig. 3). These calculations were imperative for elucidating the reaction conditions experienced by NorHA bioinks during the *in situ* crosslinking process.

With these irradiation conditions determined, photorheology experiments were performed to identify how user-defined printing parameters (capillary length, bioink flow rate, and light intensity) influenced the *in situ* crosslinking printing process. Specifically, bioink gelation profiles were created to demonstrate how the extent of reaction within the photopermeable capillary affects bioink printability. Longer capillary lengths resulted in greater ink residence times within the capillary, effectively increasing the extent of thiol-ene reaction and degree of ink crosslinking. This phenomenon was demonstrated by representative prints fabricated at variable capillary lengths (Fig. 3a). Under these printing conditions, neither 15 mm or 30 mm capillaries permitted sufficient time for stable overlaying filaments to form, resulting in unstable filament structures. While the final capillary length evaluated resulted in successful filaments (60 mm), it is important to note that if the capillary length is too long, inks may clog the capillary over time, compromising printability and giving rise to high shear forces. Clogging of the capillary could indicate interactions at the capillary interface with the hydrogel filament, which may be overcome through capillary selection or treatment of the lumen.

As expected, printing with greater bioink flow rates resulted in shorter ink residence times within the capillary and printing of unstable filaments, whereas printing with lower flow rates resulted in more precise filaments and sufficient time for the thiol-ene reaction to proceed (Fig. 3b). Lower light intensities (*I*_1_ = 5 mW/cm^2^) reduced the rate of polymerization within the capillary during printing, such that unstable filaments were formed; however, stable filaments were readily printed when *I*_1_ = 10 mW/cm^2^ and *I*_1_ = 15 mW/cm^2^ (Fig. 3c). While suitable print resolution was obtained with these print conditions at *I*_1_ = 15 mW/cm^2^, capillary clogging commonly occurred, suggesting that an upper-limit of printability exists. Thus, there is a balance between appropriate curing conditions to obtain stable filaments and the potential for clogging of the capillary with extended residence times or too rapid of crosslinking (i.e., increased light intensity).

Upon identifying permissible printing conditions via photorheology time sweeps, NorHA bioinks were printed via *in situ* crosslinking to form multilayered constructs of various shapes, including condyles and simple discs that could be used for cell culture. The process was cytocompatible, as the *in situ* crosslinking of NorHA bioinks resulted in constructs with high cell viability (>85% at 7 days after printing) and homogenously distributed MSCs. Variations in cell densities may be a concern with very long print times, but this was not an issue with the printing regimes used in the current study. There was no change in cell numbers over the first week of culture, likely due to encapsulation in the covalently crosslinked hydrogel and MSCs undergoing chondrogenesis. Further, these inks could be printed into constructs amenable to long-term culture and tissue formation. With 56 days of culture in chondrogenic media, printed constructs exhibited significant increases in compressive moduli and biochemical content associated with cartilaginous tissue. Histological analyses validated the production of both GAG and COL by encapsulated MSCs, indicating the formation and maturation of neocartilage.

An important consideration in the design of hydrogels for cartilage tissue engineering is their ability to degrade, as it has been shown that hydrogels that can readily degrade enable improved tissue formation and matrix distribution by encapsulated cells^50,51^. Since NorHA hydrogels were filled with extracellular matrix upon culture, we were unable to monitor NorHA degradation in the presence of cells; however, the elaboration of this matrix by encapsulated cells indicates that NorHA hydrogels support cartilage formation. Importantly, the degradability of NorHA hydrogels can be tuned if desired via the incorporation of degradable (e.g., matrix metalloproteinase-degradable) crosslinkers^31^. The success of this study, including printed construct stability over time, cell viability, and tissue formation, validates the approach presented here to use *in situ* crosslinking to 3D print a selected bioink. Towards translating these printed tissue constructs in the future, it will be important to consider how neocartilage may be integrated into articular focal defects for the repair of diseased cartilage. It is expected that with the development of *ex vivo* osteochondral defect models^52^ and hydrogel adhesives^53^, constructs printed via *in situ* crosslinking may be amenable to implantation.

The example presented here with the visible light crosslinking of NorHA to encapsulate MSCs towards chondrogenesis and cartilage formation is only meant to be illustrative of this printing approach. The bioink composition can be greatly varied across macromers that undergo crosslinking through light exposure, including both radical polymerizations or thiol-ene reactions in the presence of photoinitiators^54^. For example, Vega *et al*. recently developed a screening platform to identify optimal cellular environments within photocrosslinkable hydrogels^49^. Bioinks can then be readily designed from information from these types of screening platforms and implemented into the *in situ* crosslinking 3D bioprinting approach. Further, the applications of printed constructs using this approach can be easily expanded depending on the cell types and tissue of interest, and include not only for clinical applications of tissue repair, but also for *in vitro* models to probe fundamental biological questions or for drug screening. Finally, we hope the generalizable approach outlined here will be broadly accessible to numerous investigators interested in 3D bioprinting.

## Materials and Methods

### Materials

Sodium hyaluronic acid (HA, MW = 74 kDa) was purchased from Lifecore Biomedical (Chaska, MN) and lithium phenyl-2,4,6-trimethylbenzoylphosphinate (LAP) was purchased from Colorado Photopolymer Solutions (Boulder, CO). All other reagents were purchased from Sigma-Aldrich (St. Louis, MO) unless specified otherwise.

### NorHA synthesis and characterization

Sodium HA was converted into its tetrabutylammonium salt (HA-TBA) and then modified with norbornene functional groups via benzotriazole-1-yl-oxy-tris-(dimethylamino)-phosphonium hexafluorophosphate (BOP) coupling as previously described^49^. Upon dissolving HA in distilled H_2_O, Dowex 50Wx200 resin was added to the solution in a 3:1 mass ratio. After mixing for 30 minutes, the Dowex resin was filtered via vacuum filtration, and the filtrate was titrated with tetrabutylammonium hydroxide solution to a pH of 7.02–7.05. The HA-TBA solution was then frozen and lyophilized. Thereafter, 5-norbornene-2-methylamine was added to lyophilized HA-TBA and dissolved in anhydrous DMSO under inert nitrogen. BOP was then added via cannulation to the reaction round bottom flask, and the reaction was allowed to proceed for 2 hours at room temperature. The reaction was quenched with the addition of cold DI H_2_O (4 °C) and dialyzed for 5 days at room temperature. Then, the crude product solution was filtered to remove precipitates and dialyzed for an additional 3–5 days. Finally, the product was frozen and lyophilized. All synthesized polymers were stored under inert nitrogen at −20 °C and the extent of modification of HA with norbornene was quantified via ^1^H-NMR (Bruker 360 MHz, Supplementary Fig. 2). To ensure the same level of norbornene modification (~40%) was achieved across different synthesis reactions (i.e. batches), ^1^H-NMR was performed after every reaction; further, all experiments with a specific outcome were performed using the same batch of NorHA.

### Hydrogel formation and rheological characterization

One bioink formulation was investigated: 2 wt% NorHA, 0.05 wt% LAP, and 0.08 wt% DL-dithiothreitol (DTT). The absorbances of bioink components were determined using a Tecan Infinite M200 spectrometer and cuvettes with a pathlength of 1 cm. Rheological measurements were performed using an AR2000 stress-controlled rheometer (TA Instruments) fitted with a 20 mm diameter cone and plate geometry, 59 min 42 s cone angle, and 27 μm gap. The bioink formulation was placed on the rheometer and rheological properties were examined by time sweeps (1.0 Hz, 0.5% strain) in the presence of visible light (Exfo Omnicure S1500 lamp, 400−500 nm filter) applied at variable light intensities (*I*_2_, expected light intensity after attenuation through the capillary and bioink). Gelation profiles obtained from oscillatory shear time sweeps are reported as the percent of the maximum storage modulus (G′) observed after 10 minutes of irradiation with visible light.

### 3D printing of NorHA

Constructs were printed using a custom-modified 3D FDM printer (Velleman K8200) and *in situ* crosslinking at variable capillary lengths (*L* = 15–60 mm, Masterflex 96410–13), volumetric flow rates (*Q* = 0.8–3.2 mL/h), and light intensities of (*I*_1_ = 5–15 mW/cm^2^, *λ* = 400–500 nm). Upon loading inks (acellular or cellular) into a 1 mL BD syringe, Repetier software was used to slice computer-aided design (CAD) models and control the ink deposition. An Exfo Omnicure S1500 lamp with a collimating lens was used to irradiate the photopermeable capillary during material extrusion (Supplementary Fig. 1).

### Cell encapsulation and viability

All macromers were sterilized under germicidal irradiation prior to use. Primary juvenile mesenchymal stromal cells were isolated from the bone marrow of bovine femora and tibiae (Research 87, Boylston, MA) as previously described^35^. Thereafter, MSCs (P1) expanded in Dulbecco’s modified eagle medium (+10% fetal bovine serum + 1% penicillin/streptomycin) were washed, trypsinized (0.05%), centrifuged, and resuspended (20 × 10^6^ cells/mL) in NorHA dissolved in phosphate buffered saline (PBS) and manually transferred to a 1 mL BD syringe. Following 3D bioprinting, constructs were cultured in chondrogenic media (2.50 µg mL^−1^ amphotericin B, 1 × 10^−3^ M sodium pyruvate, 40 µg mL^−1^ L-proline, 1 × 10^−7^ M dexamethasone, 50 µg mL^−1^ ascorbic acid 2-phosphate, 1% ITS + , and 5 ng mL^−1^ TGF-β3). For cell viability analyses, printed hydrogels were stained with calcein AM/ethidium homodimer (0, 3, 7 days) according to manufacturer’s instructions (Invitrogen). Confocal images (Leica SP5) of stained, cell-laden constructs were analyzed using Image J software to assess both the cell viability and cell density of the top, middle, and bottom thirds of printed constructs. Cell viability was calculated as the number of live cells per total cells within a single image (n ≥ 3 gels, 9 images per group). Cell density was calculated by counting the total number of cells within randomly placed 600 × 600 *μ*m^2^ image frames (n ≥ 3 gels, 9 images per group).

### Gene expression analysis

PCR was performed for MSCs encapsulated in printed discs as previously described^55^. After 3 days of culture, samples were mechanically agitated using a handheld tissue homogenizer so that RNA could be isolated via Trizol (Invitrogen). Isolated RNA was reverse transcribed to cDNA, and PCR was then conducted on an Applied Biosystems 7300 Real-Time PCR system. Type II-collagen (COLII), aggrecan (ACAN), type I-collagen (COL I) and SOX9 were selected as targets, with glyceraldehyde 3-phosphate dehydrogenase (GAPDH) used as a housekeeping gene. Gene expression relative to MSCs expanded on tissue culture plastic was determined using the ΔΔCT method, where the fold difference was found by 2^−ΔΔC^.

### Construct mechanical and biochemical characterization

Upon printing of hydrogel bioinks (2 wt% NorHA, 0.05 wt% LAP, 0.08 wt% DTT), mechanical testing was performed (TA Instruments, DMA Q800) to determine the compressive moduli of samples. Hydrogels were secured within a fluid cup via a 0.01 N pre-load, compressed until failure at a rate of 0.5 N min^−1^, and the moduli calculated as the slope from 10–20% strain. After culture for 0, 28, and 56 days, constructs were fixed in 10% buffered formalin for 2 hours at room temperature and then washed three times with PBS. Constructs were cut into halves for either biochemical or histological analysis. Towards quantifying the biochemical content of constructs, samples were first digested via papain (0.56 U mL^−1^ in a mixture of 0.1 M sodium acetate, 10 M cysteine hydrochloric acid, and 0.05 M ethylenediaminetetraacetic acid, pH 6.0, ~1 mL/construct) at 60 °C overnight. Dimethylmethylene Blue (DMMB), PicoGreen, and hydroxyproline assays (Abcam Hydroxyproline Assay Kit, ab222941) were subsequently performed to quantify sulfated glycosaminoglycan (GAG), DNA, and collagen (COL) contents, respectively^56^.

### Construct histological characterization

To histologically analyze samples, constructs were first embedded in paraffin and incubated for 24 hours at 4 °C. Thereafter, embedded samples were sectioned (5 µm) and stained with alcian blue (1%, pH 1.0, Newcomer Supply), anti-collagen type I (COL I, mouse monoclonal anticollagen type 1, Millipore Sigma), or anti-collagen type II (COL II, mouse monoclonal anticollagen type II, Developmental Studies Hybridoma Bank) antibodies to observe GAG, COL I, and COL II, respectively. Native tissue samples were isolated from the femoral condyle of a juvenile bovine joint and processed in the same manner. To quantify staining, images were first converted to 8-bit and then inverted as previously described^55^. For each section, mean intensities for three distinct and randomly selected frames were measured in Image J.

### Statistical analysis

All data are reported as mean ± standard deviation and n ≥ 3 unless specified otherwise, and all statistics were performed using GraphPad Prism 7 software. For comparisons between two groups, Student t-tests were performed with two-tailed criteria and significance determined at p < 0.05. For comparisons between more than two groups, one-way analysis of variance (ANOVA) was performed with *post hoc* testing and significance determined at p < 0.05. Holm-Sidak correction was used for multiple comparisons with *α* = 0.05.

## Supplementary information

Supplementary Information

Supplementary Movie

## Data Availability

The authors declare that all the relevant data supporting the findings of this study are available within the paper and its supplementary information, and from the corresponding authors upon reasonable request.
