# Supplementary material for: 3D bioprinting via an in situ crosslinking technique towards engineering cartilage tissue
Source: Sci Rep. 2019 Dec 27;9:19987. doi: 10.1038/s41598-019-56117-3 (PMC6934815; doi:10.1038/s41598-019-56117-3)
Supplement: Supplementary file 1 — Supplementary Information [file 41598_2019_56117_MOESM1_ESM.docx]

**Supplementary Information**

**3D bioprinting via an *in situ* crosslinking technique towards engineering cartilage tissue**

Jonathan H. Galarraga^1^, Mi Y. Kwon^1^, Jason A. Burdick^1*^

^1^Department of Bioengineering, University of Pennsylvania, Philadelphia, PA 19104, USA

*Correspondence to J.A. Burdick (burdick2@seas.upenn.edu)

**Supplementary Figures**

**Supplementary Figure 1.** ***In situ* crosslinking technique setup.** a) Image of the 3D printing setup employed to print NorHA bioinks via *in situ* crosslinking. b) Zoomed image of collimated, visible blue light irradiation through a photopermeable capillary. NorHA bioink is extruded through a syringe, such that stable filaments are formed and deposited via CAD/controller.

**Supplementary Figure 2. ^1^H NMR characterization of NorHA in D_2_O.** Norbornene modification was determined by integrating the a) vinyl protons of norbornene (2H, $\delta$~5.8-6.3 ppm) relative to the b) methyl group of HA (3H, $\delta$~1.8-2.0 ppm) to obtain a relative norbornene modification of ~40% of the disaccharide repeat units of HA.

**Supplementary Figure 3. Influence of light absorbance on printability.** a) Absorbance measurements and b) quantified molar extinction coefficients of the LAP photoinitiator at variable concentrations (*[I]*) and light wavelengths (*λ*). c) Quantification of light attenuation across the width of the capillary (*I_W_*), due to absorbing species within the NorHA bioink (1.70 mM LAP), where *I_W_*=*I_2_* for *W*=800μm (experimental parameter in this study). Drops in light intensity: *I_1_*=5.00 mW/cm^2^ to *I_2_*=4.86 mW/cm^2^ (triangle); *I_1_*=10.0 mW/cm^2^ to *I_2_*=9.72 mW/cm^2^ (square); *I_1_*=15.0 mW/cm^2^ to *I_2_*= 14.6 mW/cm^2^ (circle).

**Supplementary Figure 4. Light attenuation through the capillary.** a) Schematic illustrating the various light intensities during *in situ* crosslinking, including directly from the light guide (*I_0_*), at the inner edge of the capillary lumen (*I_1_*), after passing through the capillary lumen (*I_2_*), and at the opposite side of the capillary (*I_3_*). An equation describing this relationship is shown to account for light attenuation by the capillary itself. b) Experimental measurements of the incident light intensity (*I_0_*) compared to the light intensity at the opposite end of the capillary from the light source (*I_3_*). c) Calculated calibration curve to determine the required *I_0_* to achieve a desired *I_1_* to cure the bioink.

**Supplementary Figure 5.** **Rheological properties of bioinks across various light intensities**. Representative photorheology time sweeps (1 Hz, 0.5% strain) during the photocrosslinking of the NorHA bioink with visible light (400-500 nm) at either a) *I_1_*=5 mW/cm^2^ or b) *I_1_*=15 mW/cm^2^, illustrating increases in storage (G’, closed circles) and loss (G”, open circles) moduli over time.

**Supplementary Figure 6.** **Stability of discs printed via *in situ* crosslinking technique**. Printed discs were immersed in PBS for 0, 3, or 7 days and imaged to quantify any changes in the disc diameter over time. n≥3, n.s. = not significant.


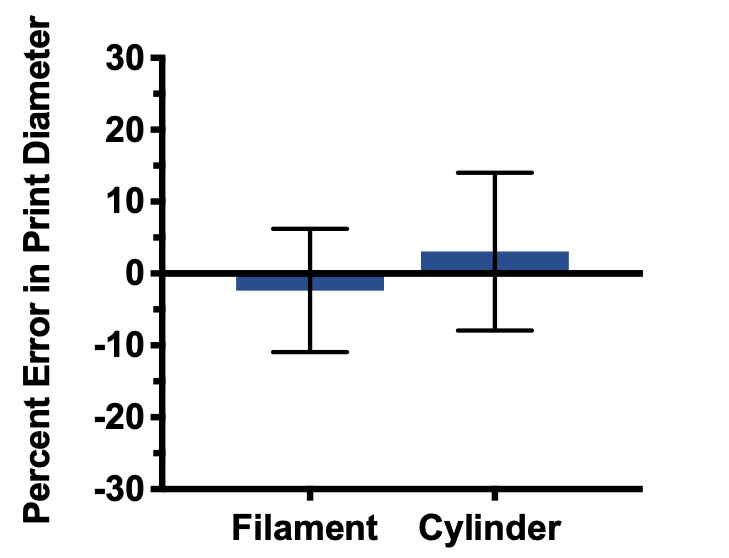


**Supplementary Figure 7.** **Quantification of printing error associated with *in situ*** **crosslinking technique**. Percent errors between target dimensions and observed dimensions are reported for both printed filaments (target dimension: 800 μm diameter) and printed discs (target dimension: 6.3 mm diameter). n≥7 constructs.


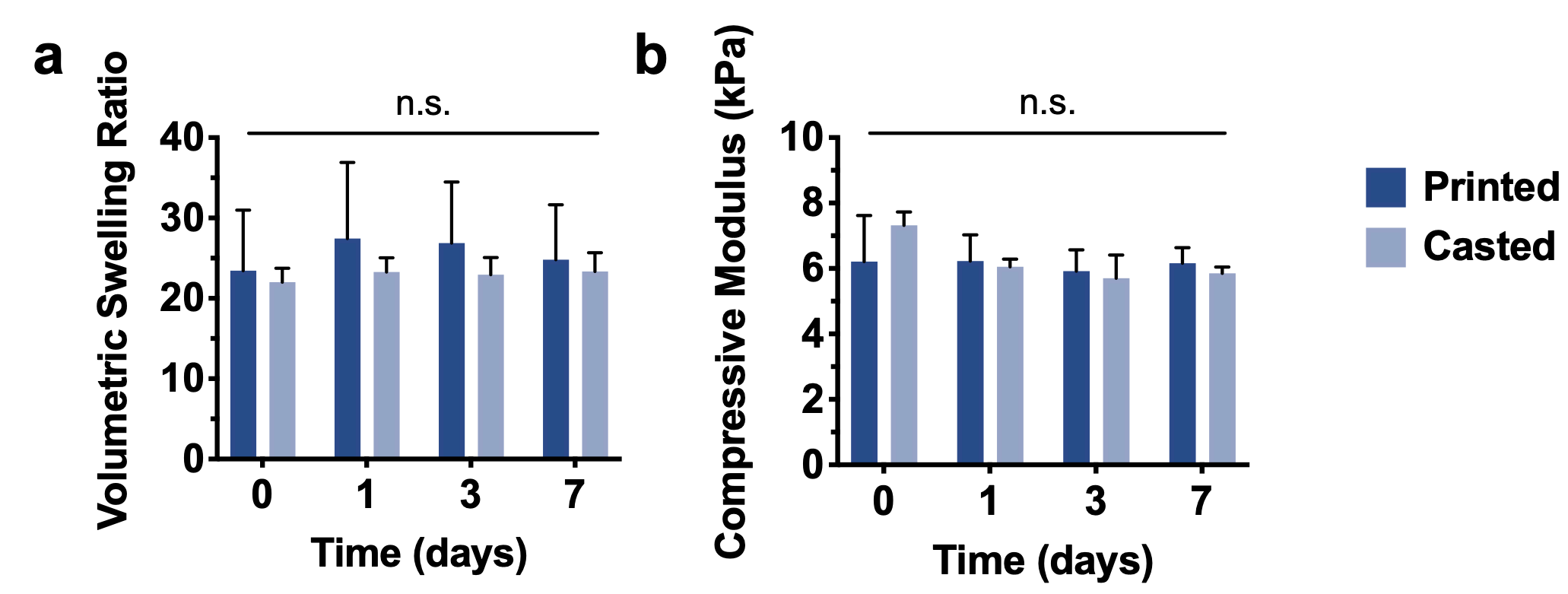


**Supplementary Figure 8.** **Swelling behavior and mechanics of printed versus casted NorHA discs**. a) Volumetric swelling ratios are reported for printed and casted discs as the ratio between hydrogel wet weights and dry weights. Samples were incubated in PBS and analyzed at days 0,1,3 and 7. b) Compressive moduli for printed and casted discs at days 0,1,3 and 7. n≥3. n.s. = not significant.

**Supplementary Figure 9.** **Relative gene expression of encapsulated MSCs in printed NorHA constructs**. Mean fold difference of type II-collagen (COLII), aggrecan (ACAN), type I-collagen (COLI) and SOX 9 gene expression for printed discs cultured for 3 days relative to MSCs cultured on tissue culture plastic (i.e. day 0, 2D control). Dashed line represents expression level of control group, which are cells at the time of encapsulation. n≥5 printed discs, *p<0.05.
